# The Non-Coding RNA Ontology (NCRO): a comprehensive resource for the unification of non-coding RNA biology

**DOI:** 10.1186/s13326-016-0066-0

**Published:** 2016-05-04

**Authors:** Jingshan Huang, Karen Eilbeck, Barry Smith, Judith A. Blake, Dejing Dou, Weili Huang, Darren A. Natale, Alan Ruttenberg, Jun Huan, Michael T. Zimmermann, Guoqian Jiang, Yu Lin, Bin Wu, Harrison J. Strachan, Yongqun He, Shaojie Zhang, Xiaowei Wang, Zixing Liu, Glen M. Borchert, Ming Tan

**Affiliations:** School of Computing, University of South Alabama, Mobile, Alabama, 36688-0002 USA; Department of Biomedical Informatics, University of Utah, Salt Lake City, 84112-5775 Utah USA; Department of Philosophy, University at Buffalo, Buffalo, 14260-4150 New York USA; Genome Informatics, The Jackson Laboratory, Bar Harbor, Maine, 04609-1523 USA; Computer and Information Science Department, University of Oregon, Eugene, Oregon, 97403-1202 USA; Miracle Query, Inc., Eugene, Oregon, 97403-1202 USA; Department of Biochemistry and Molecular & Cellular Biology, Georgetown University Medical Center, Washington D.C., 20007-1485 USA; School of Dental Medicine, University at Buffalo, Buffalo, 14214-8006 New York USA; Department of Electrical Engineering and Computer Science, University of Kansas, Lawrence, 66047-7621 Kansas USA; Health Sciences Research, Division of Biomedical Statistics and Informatics, Mayo Clinic College of Medicine, Rochester, 55905-0001 Minnesota USA; Center for Computational Science, University of Miami, Miami, 33146-2960 Florida USA; Department of Microbiology & Immunology, First Affiliated Hospital, Kunming Medical University, Kunming, Yunnan, 650032 China; Department of Microbiology & Immunology, University of Michigan Medical School, Ann Arbor, Michigan, 48109-5624 USA; Department of Computer Science, University of Central Florida, Orlando, 32816-2362 Florida USA; Department of Radiation Oncology, Washington University School of Medicine, St. Louis, Missouri, 63110-0001 USA; Mitchell Cancer Institute, University of South Alabama, Mobile, Alabama, 36604-1405 USA; Department of Biology, University of South Alabama, Mobile, Alabama, 36688-0002 USA

**Keywords:** Non-coding RNA, Biomedical ontology, Domain ontology, Reference ontology, Ontology development, Data annotation

## Abstract

In recent years, sequencing technologies have enabled the identification of a wide range of non-coding RNAs (ncRNAs). Unfortunately, annotation and integration of ncRNA data has lagged behind their identification. Given the large quantity of information being obtained in this area, there emerges an urgent need to integrate what is being discovered by a broad range of relevant communities. To this end, the Non-Coding RNA Ontology (NCRO) is being developed to provide a systematically structured and precisely defined controlled vocabulary for the domain of ncRNAs, thereby facilitating the discovery, curation, analysis, exchange, and reasoning of data about structures of ncRNAs, their molecular and cellular functions, and their impacts upon phenotypes. The goal of NCRO is to serve as a common resource for annotations of diverse research in a way that will significantly enhance integrative and comparative analysis of the myriad resources currently housed in disparate sources. It is our belief that the NCRO ontology can perform an important role in the comprehensive unification of ncRNA biology and, indeed, fill a critical gap in both the Open Biological and Biomedical Ontologies (OBO) Library and the National Center for Biomedical Ontology (NCBO) BioPortal. Our initial focus is on the ontological representation of small regulatory ncRNAs, which we see as the first step in providing a resource for the annotation of data about all forms of ncRNAs. The NCRO ontology is free and open to all users, accessible at: http://purl.obolibrary.org/obo/ncro.owl.

## Introduction

It is known that non-coding RNAs (ncRNAs), a special class of functional RNA molecules, will not be translated into proteins. The chemical identity and first guesses as to the role of RNA were discussed by Casperson and Schultz back in 1939, and the first RNA structure was reported by Alexander Rich in 1956 [1]. Since then, many types of ncRNAs have been identified, including the now well-known transfer RNAs (tRNAs) and ribosomal RNAs (rRNAs), in addition to the more recently discovered long non-coding RNAs (lncRNAs), microRNAs (miRNAs), and so forth. Many ncRNAs perform important roles in the realization of a wide range of molecular functions as well as in affecting many different biological and pathological processes. As such, interest in ncRNA biology has grown throughout biomedicine, biomedical informatics, and clinical sciences. In addition, the fertile area of ncRNA research has been significantly enhanced in recent years by new sequencing technologies that have generated continuously increasing quantities of available data. However, annotation and integration of data about ncRNAs, the functions regulated by ncRNAs for example, has lagged behind their identification, resulting in an urgent need for effective methodologies to bring together discoveries continuously deriving from different segments of the ncRNA research community.

Emerging semantic technologies provide computational methodologies that promote more precise communication among scientists, enable more effective information retrieval and integration across diverse resources, and extend the power of computational technologies to perform data exploration, inference, and mining [2–7]. In particular, the sorts of reasoning (inference) enabled by semantic technologies are not available where we are confined to traditional relational database systems or text-based search and query. By placing more emphasis on the semantics (i.e., the intended meaning) of data, semantic technologies and domain ontologies enable us to establish more meaningful connections among original data, thereby helping to bridge gaps in our knowledge. Moreover, semantic data connections are established in a highly flexible manner that allows these connections to be much more easily extended — for example when new sorts of entities are discovered — than is possible using more traditional approaches.

Among all successful efforts in applying semantic technologies in the biomedical domain, the Open Biological and Biomedical Ontologies (OBO) Library [8] is of special importance in that it has served as an umbrella for different ontologies shared across various biological, biomedical, and clinical domains. However, there has until now existed in the OBO Library no comprehensive ontologies specifically designed for the ncRNA domain, although portions of the domain are catalogued in several orthogonal ontologies. The National Center for Biomedical Ontology (NCBO) BioPortal [9], a repository of biological and biomedical ontologies (short for bio-ontologies), is another effort in some ways parallel to the OBO Library but with a broader scope and lower hurdles for admission. However the BioPortal, too, contains, no comprehensive ncRNA ontologies. These observations indicate that there is an important gap that needs to be filled — hence the Non-Coding RNA Ontology (NCRO) project. As the *first* comprehensive, domain-specific ontology in the ncRNA field, the NCRO ontology aims to supply a systematically structured, precisely defined controlled vocabulary for the ncRNA domain, consisting of a set of common, standardized terms and relations that will facilitate the discovery, curation, analysis, exchange, and reasoning of data about the structures, functions, and molecular, cellular, organismal, therapeutic, or biotechnological uses of ncRNAs. The NCRO ontology can serve as a resource for annotating and integrating ncRNA data produced by diverse communities, thereby significantly enhancing integrative and comparative analysis of the myriad resources currently housed in disparate sources. We believe that the NCRO will help to address a vital need for the comprehensive unification of ncRNA biology. We aim to integrate genomic and sequence-based annotation with gene expression regulation, secondary and 3D structure information, protein interactions, and their inter-relationships. Our initial focus is on the ontological representation of small regulatory ncRNAs, which we see as the first step in providing a standardized resource for (1) annotating data about all forms of ncRNAs and (2) facilitating knowledge capture in the ncRNA domain.

The rest of this paper is organized as follows. Section ‘Related work’ summarizes state-of-the-art research in ncRNAs and bio-ontologies; Section ‘Ontology scope’ gives an overview of the scope covered by the NCRO ontology; Section ‘Ontology development’ introduces NCRO development principles and procedure; Section ‘NCRO terms, relations, and reasoning’ describes NCRO terms and relations, as well as ontology reasoning; Section ‘Examples in NCRO annotations’ presents two examples to demonstrate how NCRO annotations and ontology reasoning can be performed to facilitate knowledge capture; finally, Section ‘Conclusions’ concludes with future research directions.

## Related work

### Related work in ncRNA research

Prior research, [10–12] for example, has uncovered numerous ncRNA genes, and recent advances in next generation sequencing technology have resulted in an even greater number and faster pace of discovery of ncRNA genes. In fact, Nature has a whole site dedicated to key apes in this area [13]. Given the relatively large proportion of the genome dedicated to ncRNA genes, significant potential exists to explore ncRNAs that may have diverse biological roles.

Abnormal expression of some ncRNAs is involved in human disease. For example, alterations of gene-regulatory ncRNA expression are involved in the development, progression, and metastases of human cancer [14]. When differentially expressed gene-regulatory ncRNAs play roles in altering target gene expression, further phenotypic effects can be realized. Differential expression of such ncRNAs in malignant versus normal tissue can be exploited as a biomarker used for diagnosis, prediction of patient outcome, or monitoring the effectiveness of cancer therapeutics. Therefore, these gene-regulatory ncRNAs are potential therapeutic targets for cancer therapy. In recent years serious attempts have been made to effectively deliver ncRNA into tumors in animal models. Some of the attempts have already shown promising therapeutic efficacy [15–17]. In RNA interference therapy and drug development, a first-in-human trial has been conducted in cancer patients who were administered with lipid nanoparticles (LNP) formulated siRNA targeting VEGF and KSP [18].

Aberrant expression of ncRNAs has been associated with not only cancers but also numerous other diseases, including autism, hearing loss, Alzheimer’s disease, Prader-Willi Syndrome, diabetes, and psoriasis [19–23]. Tissue-specific miRNAs have been shown to be involved in cardiovascular, muscular, and neurodegenerative diseases, and pharmaceutical companies are developing new therapeutic molecules that alter the function or expression of specific miRNAs for treating these and other human diseases [24].

### Related work in bio-ontologies

There are several pre-existing bio-ontologies that are relevant to the development of an ontology in the domain of functional non-coding RNA. The RNA Ontology (RNAO) [25] is a reference ontology created to catalogue the molecular entities composing primary, secondary, and tertiary components of RNA. The goal of the RNAO project is to enable integration and analysis of diverse RNA datasets. The Gene Ontology (GO) [26] is by far the most successful and widely used bio-ontology, consisting of three independent sub-ontologies: biological processes, molecular functions, and cellular components. The GO has been utilized to annotate both protein and RNA gene products across multiple organisms. The Sequence Ontology (SO) [27] is an ontology that is designed to capture genomic features and the relationships that obtain between them. This ontology contains the features necessary to annotate a genome sequence with structural features such as gene models and also the terms necessary for the annotation of the location and extent of genomic variants. The PRotein Ontology (PRO) [28] has been developed with a particular focus on human proteins and disease-related variants thereof, providing an ontological representation of proteins. As proteins are often the functional entities in the processes impacted by the regulatory effect of ncRNAs, they are an important factor in the understanding of ncRNA. The Ontology for MIcroRNA Target (OMIT) [29–31] is a miRNA domain ontology that is being developed as part of the OmniSearch project. The purpose is to establish standard metadata in miRNA domain for more effective identification of the roles of miRNAs in various human diseases. The ontology of Chemical Entities of Biological Interest (ChEBI) [32] provides the terminology and relationships to describe small molecules.

There are also other bio-ontologies that are in use in a wider context that are also important for the description of clinical impact of ncRNA. SNOMED CT [33] is a comprehensive, clinically oriented medical terminology system, and also a reference standard in the United States Meaningful Use program that promotes the use of certified electronic health record (EHR) technology to improve quality, safety, and efficiency, as well as to reduce health disparities [34]. SNOMED CT is owned and maintained by the International Health Terminology Standard Development Organization (IHTSDO). Anatomy description has been unified over multiple species with the Uberon anatomical Ontology [35]. This ontology relates taxon-specific anatomies and is fully integrated with other bio-ontologies such as the GO. The Human Disease Ontology (DOID) [36] encapsulates the terminology of diseases and provides equivalent mappings to many related terminologies. The NCI Thesaurus (NCIt) [37] is a reference biomedical ontology published by the National Cancer Institute (NCI) with terminology that includes clinical care, translational and basic research, and public information and administrative activities.

Additionally, there are ontologies that address the domain of data collection and are pertinent to the understanding of ncRNA. An ontology that covers the domain of translational research is the Ontology of Biomedical Investigations (OBI) [38], describing the foundational terminology needed to define experimental processes and investigation. Moreover, the Information Artifact Ontology (IAO) [39] arose as a branch of OBI, to define the foundational entities of scientific information in the digital domain.

Note that all bio-ontologies described in this section except for SNOMED CT are included in both the OBO Library and NCBO BioPortal. SNOMED CT is included only in the BioPortal.

## Ontology scope

The NCRO ontology will represent:

1. All known subtypes of ncRNA molecules including those created in living organisms as well as those engineered or adapted for some purposes (aptamers for example [40]) — this aspect will utilize high-level terms defined in both the SO and ChEBI, with more specific terms defined in the NCRO;
2. The structure involved in each ncRNA type, including sequence and conformation — this aspect will utilize the RNAO;
3. The functions, dispositions, and roles of ncRNAs, as well as the processes in which these are realized^1^ — this aspect will utilize, mostly, the GO, with gaps specific to ncRNAs filled by the NCRO or other ontologies;
4. Different clinical phenotypes associated with expression of normal and/or abnormal ncRNAs — this aspect will utilize the SNOMEDCT, NCIt, and Human Disease Ontology (DOID); and finally,
5. Various relations that are unique to ncRNAs and their different components.

The initial focus of our work in building the ontology is on small regulatory ncRNAs. Nevertheless, we have designed an overarching framework of high-level terms for other ncRNAs, such as: circular RNA (circRNA), lncRNA, rRNA, small interfering RNA (siRNA), small nuclear RNA (snRNA), and tRNA. These high-level terms, all of which are direct child terms of the term “ncRNA,” serve as placeholders: a more detailed hierarchy underneath each term, along with relevant relations, will be developed at a later project stage.

## Ontology development

### Development principles

In the development pipeline for the NCRO ontology, we have observed a set of practices proposed by the OBO Foundry Initiative [41, 42]. Above all, the ontology should be: freely available; expressed in a standard language; documented for successive versions; orthogonal to existing ontologies; including natural language specifications; developed collaboratively; and used by multiple researchers.

### Compliance with established upper-level ontologies

All NCRO terms descend from terms defined in the Basic Formal Ontology (BFO) v2.0 [43]. The BFO is a small, upper-level ontology that is designed for use in supporting information retrieval, analysis, and integration in scientific and other domains. Because the BFO is a well-established upper ontology adopted by all OBO ontologies, our strategy to make the NCRO a BFO-compliant ontology will set the stage for interoperability between the NCRO ontology and other currently existing OBO ontologies.

As for relations, besides those defined in the NCRO, we have also used a set of well-defined relations in the Relation Ontology (RO) [44, 45], such as: “*part of*,” “*participates in*,” and “*precedes*,” all of which relate different types defined in the BFO. Greater details of various relations can be found in Section ‘NCRO terms and relations’ and Table 3.

### Strategy for orthogonality

Out of the set of OBO Foundry principles, orthogonality is of special importance in defining the novelty of the NCRO ontology. Our strategy to abide by this principle is that we have imported and reused extant terms wherever possible, focusing especially on terms from OBO ontologies, SO, GO, PRO, and ChEBI for example. Such terms have been imported with their original identifier information using internationalized resource identifiers (IRIs)/uniform resource identifiers (URIs). This strategy helps us to achieve the maximum possible orthogonality. Table 1 demonstrates a subset of imported terms. More details can be found in Section ‘NCRO terms, relations, and reasoning,’ where percentages of imported terms from various existing bio-ontologies are calculated.

**Table 1:** A subset of terms imported into the NCRO ontology

Imported term			Source Ontology			Original ID
miRNA			Sequence Ontology			SO:0000276
ncRNA			Sequence Ontology			SO:0000655
small_regulatory_ncRNA			Sequence Ontology			SO:0000370
gene			Sequence Ontology			SO:0000704
promoter			Sequence Ontology			SO:0000167
binding			Gene Ontology			GO:0005488
transcription,			Gene Ontology			GO:0006351
DNA-templated						
translation			Gene Ontology			GO:0006412
metabolic_process			Gene Ontology			GO:0008152
protein			PRotein Ontology			PR:000000001
organism			Ontology for			OBI:0100026
			Biomedical Investigations			
cell			Gene Ontology			GO:0005623
cell line			Cell Line Ontology			CLO:0000031
molecular entity			Chemical Entities of			CHEBI:23367
			Biological Interest Ontology			
organ			Uber Anatomy Ontology			UBERON:0000062
tissue			Uber Anatomy Ontology			UBERON:0000479
disease			Human Disease Ontology			DOID:4

### The NCRO team and domain expertise

The NCRO team members come from a wide variety of communities, covering computer science, ontology engineering, wet-lab biological research, biomedical informatics, and clinical sciences. The wide scope of participants will provide (1) the necessary expertise in ontology development and ontology-based reasoning and (2) the ncRNA domain knowledge including expertise in ncRNA-relevant phenotype. It will also help to ensure (3) a diversity of communities eager to adopt the NCRO ontology for use in representing and annotating ncRNA data.

### Dynamic ontology construction procedure

The NCRO development is from the top down (starting with more general terms), progressively utilizing the ncRNA domain knowledge provided by the cellular biologists and clinical investigators in the project team. Lower levels of the ontology were then further developed on the basis of a thorough analysis of representative ncRNA-related databases (Table 2). Moreover, an iterative procedure, including a series of interviews, exchanges of documents, refinements, and related documentations, is being followed to make the NCRO a dynamic ontology. In addition to a dedicated project website [46], we have utilized GitHub [47] to further assist the management and version control of the ontology during both design and implementation, including an established issue tracker [48] to facilitate discussion among the members of an open group of investigators, so that OBO Foundry principles can be better followed.

**Table 2:** A list of ncRNA-related databases

Database name	Brief introduction	Web link
Ensembl ncRNA	A database of ncRNA annotations.	http://www.ensembl.org/info/genome/genebuild/ncrna.html
GENCODE	A database for annotation of gene features.	http://www.gencodegenes.org
lncRNAdb	A reference database for functional lncRNAs.	http://www.lncrnadb.org
lncRNAtor	A Web portal encompassing lncRNA data.	http://lncrnator.ewha.ac.kr
miRBase	A database of miRNA sequences and annotation.	http://www.mirbase.org/
NDB	A database of experimentally determined nucleic acids.	http://ndbserver.rutgers.edu
NONCODE	A database of ncRNAs except for tRNAs and rRNAs.	http://www.noncode.org/
NRED	An ncRNA expression database.	http://nred.matticklab.com/cgi-bin/ncrnadb.pl
Rfam	A database of a collection of RNA families.	http://rfam.xfam.org
RMDB	Chemical Mapping Data of RNA Sequences.	https://rmdb.stanford.edu

### Naming conventions

Each NCRO term has a unique identifier consisting of a prefix and seven digit numerical string, as in: NCRO_0000001. On the other hand, each NCRO term is also assigned a human-readable label. We have followed a set of OBO Foundry naming conventions [49] to design such labels. Specifically:

- Labels are written in lower cases except for commonly accepted acronyms such as “RNA” and “ncRNA.”
- Hypens are kept as is if they are commonly used in, or easily understood by, the ncRNA community, as in: “hsa-miR-125b.”

For greater readability, we *italicize* all relations throughout this paper, whether they are defined in the NCRO or imported from the RO and BFO.

### Ontology languages and development tools

We have chosen both the Web Ontology Language (OWL) [50] and OBO formats to describe the ontology: both are widely accepted in OBO Foundry community and the former is recommended by the World Wide Web Consortium (W3C). A first version of the ontology was authored in OBO-Edit [51] and translated to OWL by the ROBOT tool [52]; then the OWL version has been subsequently edited. Moving forward, our focus will be placed on editing and releasing the OWL version to take advantage of OWL-specific features such as availability of ontology reasoners and triple stores, as well as enhanced annotation expressivity.

## NCRO terms, relations, and reasoning

### NCRO terms and relations

The current version NCRO (http://purl.obolibrary.org/obo/ncro.owl) is our first production release. There are a total of 3,078 terms and 27 relations (besides a total of 5,394 *is_a* relations). Terms break down as follows: 82.68 % were defined in the NCRO ontology itself, and the rest were imported from extant ontologies: BFO (1.14 %), GO (8.67 %), SO (6.50 %), PRO (0.10 %), CHEBI (0.29 %), OBI (0.13 %), IAO (0.06 %), DOID (0.13 %), CLO (0.06 %), and UBERON (0.16 %). As for relations, many (55.56 %) were imported from the RO, and the rest (51.03 %) were defined in the NCRO.

Orthogonality among different ontologies has been widely accepted in the bio-ontology community. To achieve better orthogonality, it is a common practice to reuse contents defined in relevant, existing ontologies. This is our motivation to import terms and relations from extant ontologies, as demonstrated above. On the other hand, it is not trivial to obtain 100 % orthogonality, because ontologies are continuously being developed for good reasons within specific domains and by different groups. As a result, given the holistic nature of biology, along with the fact that different applications most likely have adopted different development methodologies and have focused on various emphases, there will inevitably be some overlaps among ontologies regarding their covered terms and/or relations. For example, the term “analgesic_treatment” defined in the NCRO^2^ is similar with the term “analgesic treatment” defined in the Malaria Ontology^3^. Such overlaps may have negative impacts on logical inferencing if ontology reasoning is performed across relevant ontologies. Whereas it is not realistic, if not impossible at all, to obtain “pure” (i.e., 100 %) orthogonality, one effective way to handle this situation is to add cross-references in the ontologies.

**Fig. 1:**
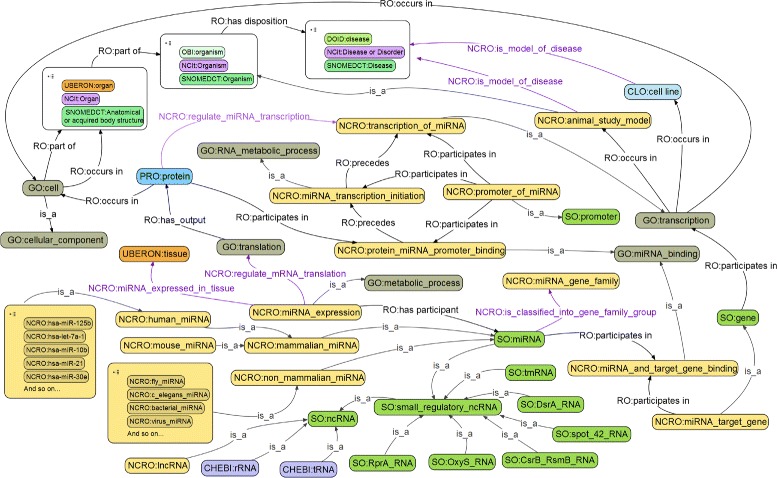
NCRO core terms and relations. The design of core terms and relations in the NCRO ontology (terms and relations are represented in the format of “PREFIX:label”)

Details of all terms and relations in the NCRO ontology are publicly available [46, 47]. In addition, Table 3 presents a subset of relations defined in or imported into the NCRO; and Fig. 1 shows a complete view of the core portion designed in the ontology, using the format of “PREFIX:label” to describe each term or relation.

**Table 3:** A subset of relations defined in or imported into the NCRO ontology

Relation	Domain	Range	Explanation
*NCRO:is_classified_into*	miRNA	miRNA_gene_family	Each miRNA can be classified
*_gene_family_group*			into some gene family.
*NCRO:miRNA_expressed*	miRNA_expression	tissue	miRNAs can be expressed in some
*_in_tissue*			specific tissues.
*NCRO:regulate_mRNA*	miRNA_expression	translation	miRNAs can regulate the translation
*_translation*			process of some mRNAs.
*NCRO:regulate_miRNA*	protein	transcription_of_miRNA	Proteins can regulate the
*_transcription*			transcription process of some miRNAs.
*NCRO:is_model_of_disease*	material entity	disease	A cell line is a model of some disease.
*RO:participates in*	gene	transcription,	A gene participates in the regulation
		DNA-templated	of some transcription.
*RO:participates in*	miRNA_target_gene	miRNA_and_target_	A link to connect a miRNA and its
	miRNA	gene_binding	likely target gene.
*RO:participates in*	promoter_of_miRNA	protein_miRNA_	Connection between the miRNA
	protein	promoter_binding	promoter and protein.
*RO:precedes*	protein_miRNA_	miRNA_	Protein-promoter binding happens
	promoter_binding	transcription_initiation	before the transcription.
*RO:precedes*	miRNA_transcription_	transcription_of_miRNA	Transcription initiation happens before
	initiation		the transcription process.
*RO:part of*	organ	organism	An organ is part of some organism.
*RO:part of*	cell	organ	A cell is part of some organ.

### Ontology reasoning

The NCRO ontology provides a standardized, well-structured, and precisely defined set of terms, along with various relations among these terms. The NCRO thus:

- Enables machine-readable description and encoding of ncRNA annotations — so that these annotations can be identified and integrated in a more precise and effective manner.
- Helps establish connections among diverse ncRNA-related data sources — through cross-references that are formally defined in the expert-built NCRO ontology and other extant domain bio-ontologies.
- Provides necessary software substrates for automated ontology reasoning — (1) annotated data can be more readily verified through validating internal consistency and (2) further, insights for new discoveries can be effectively derived through inferred relations and more expressive queries.

In this way, the NCRO ontology assists in establishing ncRNA common data elements and data exchange standards. Consequently, it will greatly enhance data sharing and exchange as well as comparative analysis on ncRNA annotations from heterogeneous sources. In addition, because the NCRO ontology covers species other than Homo sapiens, it will enable communication among different model organism groups. The next section contains greater details on how NCRO-based ontology reasoning can further facilitate ncRNA knowledge capture.

## Examples in NCRO annotations

**Fig. 2:**
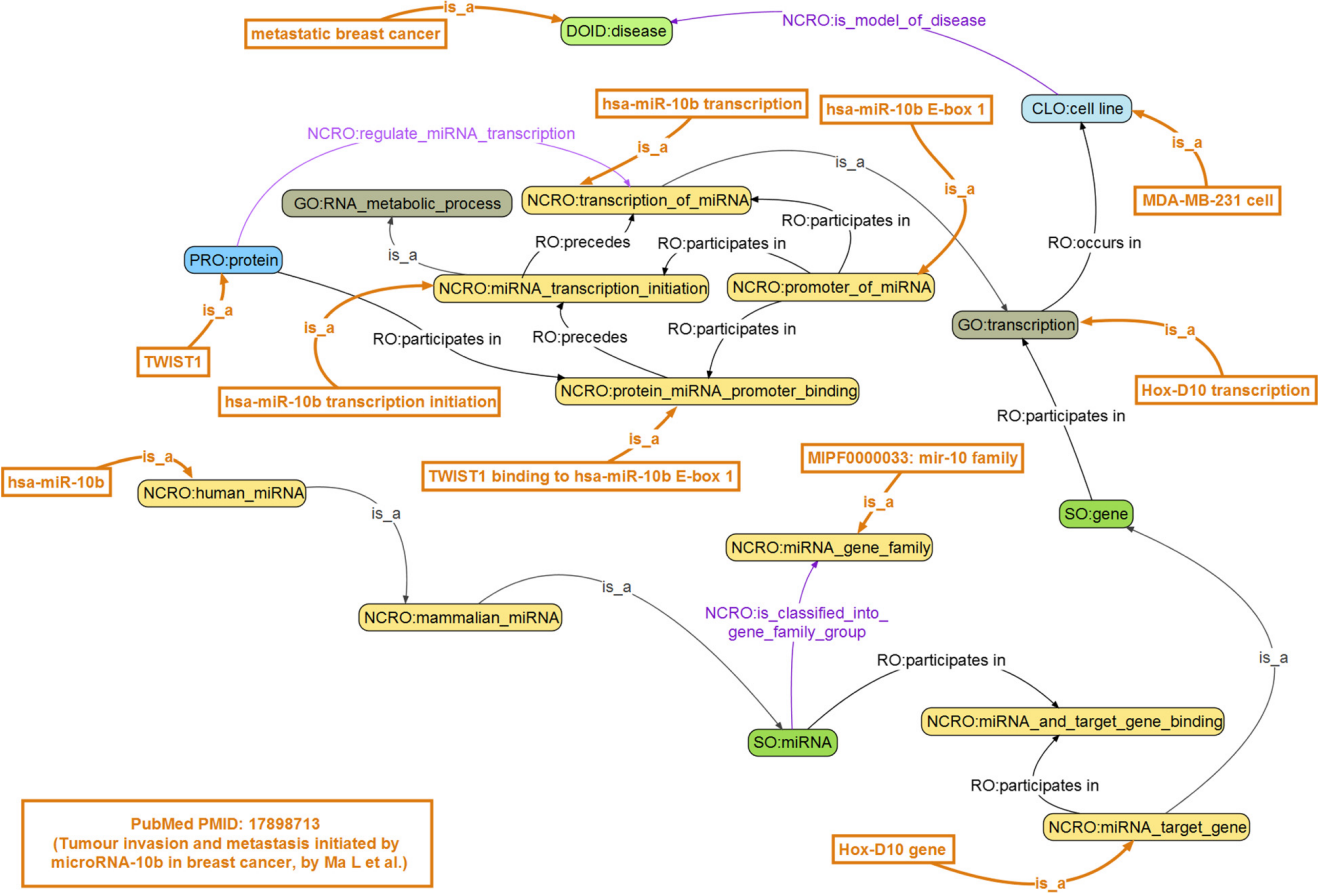
Step one in use case I. NCRO Annotation Use Case I (Step One — ontology-based annotation)
- Hox-D10 transcription is found in MDA-MB-231 cell line, which is a model of metastatic breast cancer.
- The transcription of hsa-miR-10b itself is found in MDA-MB-231 cell line.
- The details of hsa-miR-10b transcription process are: (1) a protein, TWIST1, binds to E-box1, a promoter of hsa-miR-10b; (2) such a binding leads to an initiation of hsa-miR-10b transcription; and finally, (3) hsa-miR-10b transcription occurs in MDA-MB-231 cell line under the regulation of TWIST1.

We describe below two examples designed to demonstrate how NCRO annotations and NCRO-based ontology reasoning can be performed. The first example is based on important findings reported in Ma et al. (shown in Fig. 2, bottom-left corner):

- hsa-miR-10b binds to its target, Hox-D10 gene, which participates in regulation of Hox-D10 transcription.

These findings can be annotated with NCRO terms and relations, and the annotation result is shown in Fig. 2. Note that for reasons of clarity only relevant terms and relations are shown. Appropriate ontology terms were related to the above-mentioned findings, demonstrated as a set of leaf nodes in bold, brown color in the figure. For example, **Hox-D10 gene** was annotated with the term “NCRO:miRNA_target_gene,” **hsa-miR-10b E-box 1** was annotated with the term “NCRO:promoter_of_miRNA,” and **metastatic breast cancer** was annotated with the term “DOID:disease.” It is evident that the original, human-readable information contained in the paper was precisely annotated and converted into NCRO-compliant, machine-understandable knowledge, which can be readily represented in appropriate computer-friendly formats, resource description framework (RDF) triples for example.

Next, ontology reasoning can be performed on such machine-understandable knowledge to not only automatically verify the encoded knowledge but also — equally importantly — infer new knowledge that was originally hidden and inexplicit in the raw data. For example:

1. Reasoning based on the *is_a* hierarchy from **hsa-miR-10b** all the way to “SO:miRNA” as well as reasoning based on the *participates in* relation will lead to Conclusion 1: **hsa-miR-10b** binds to its target **Hox-D10 gene**.
2. Similarly, another conclusion can be readily obtained as well, that is, Conclusion 2: “**Hox-D10 gene** participates in **Hox-D10 transcription**.”

**Fig. 3:**
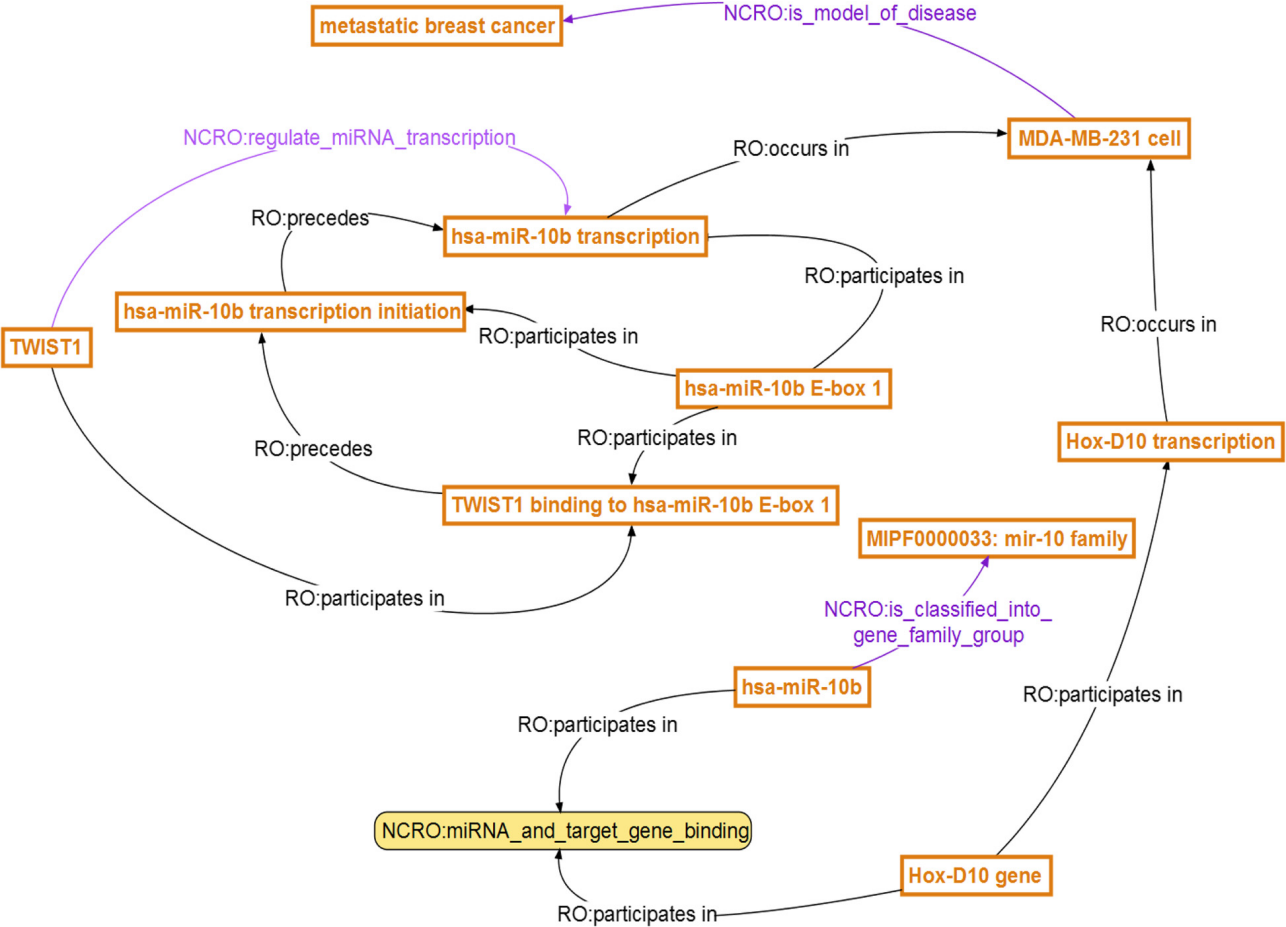
Step two in use case I. NCRO Annotation Use Case I (Step Two — ontology reasoning)

Figure 3 demonstrates the conclusions obtained following such reasoning mechanisms. In this example, based on a piece of domain knowledge defined in the NCRO ontology, “the gene family group of hsa-miR-10b is MIPF0000033: mir-10 family,” we can infer a **new hypothesis that the mir-10 family is likely to participate in the control and regulation of metastatic breast cancer disease process**. Note that this hypothesis, drawn by ontology reasoning, was not explicitly stated in the original paper; at the same time, it provides an important clue to both cancer biologists and clinical investigators for wet-lab experiment design and treatment planning, respectively. As discussed earlier in Section ‘Introduction,’ these reasoning mechanisms are not possible in any traditional relational database systems or conventional text-based search and query. In fact, this is one of the reasons why semantic technologies and domain ontologies have been playing increasingly important roles in biological and biomedical knowledge capture — by placing more emphasis on the semantics (i.e., the intended meaning) of data, semantic technologies and domain ontologies enable us to establish newly discovered, more meaningful connections among original data, which in turn help to bridge gaps in human knowledge.

**Fig. 4:**
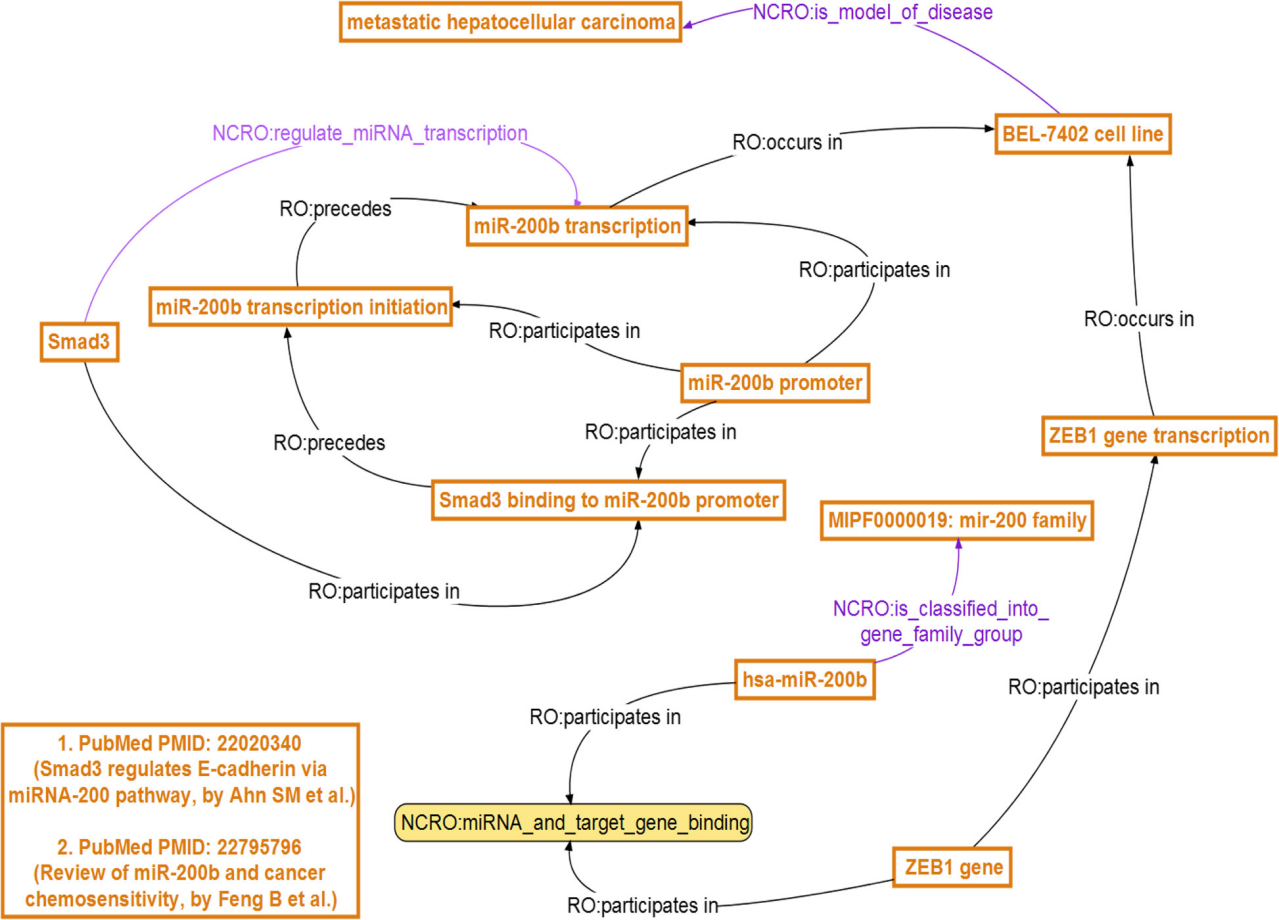
Step two in use case II. NCRO Annotation Use Case II (Step Two — ontology reasoning)

A second annotation example is demonstrated in Fig. 4: **hsa-miR-200b** is shown to be closely related to **metastatic hepatocellular carcinoma**; similarly, we can infer a new hypothesis that the mir-200 family is likely to participate in the control and regulation of metastatic hepatocellular carcinoma.

## Conclusions

Prior research has indicated that ncRNAs perform important roles in realizing a wide range of molecular functions and affecting many different biological and pathological processes. Interest in ncRNA biology has therefore grown throughout biomedicine, biomedical informatics, and clinical sciences in recent years. Due to the lack of standardized ncRNA nomenclature, there exist significant barriers to the representation, acquisition, integration, and comparison of ncRNA data. Thus, the establishment of common data elements and data exchange standards for the ncRNA domain is an important need. The OBO Library has successfully served as an umbrella for different communities of ontologists drawn from a variety of biomedical and clinical domains. Until now, however, the OBO Library contained no ontologies designed for the ncRNA domain. Likewise, the NCBO BioPortal lacked such ontologies. We developed the NCRO ontology to fill this important gap. The NCRO aims to provide a systematically structured, precisely defined ncRNA controlled vocabulary, including a set of common, standardized terms and relations, to facilitate the discovery, curation, analysis, exchange, and reasoning of data about ncRNA structures, functions, and uses. The ultimate goal of the NCRO project is to establish a virtual center to further facilitate knowledge capture about all forms and uses of ncRNAs.

In this paper, we introduced the scope, development process, and core terms and relations in the NCRO ontology. We also discussed reasoning mechanisms to further facilitate ncRNA data management, including data annotation, analysis, comparison, and integration. The examples provided showcase how NCRO annotations and NCRO-based ontology reasoning can be performed to assist cellular biologists, bioinformaticians, and clinical investigators in ncRNA-related knowledge acquisition and discovery. As a common resource for annotations of diverse ncRNA research, ***the NCRO ontology can perform an important role in the comprehensive unification of ncRNA biology***. This unification integrates genomic and sequence-based annotation with gene expression regulation, secondary and 3D structure information, protein interactions, and their inter-relationships, using standardized ontological representations. The current version of the NCRO ontology contains a total of 3,078 terms and 27 relations (besides a total of 5,394 *is_a* relations). The ontology files and design documentations are publicly available at: OBO Library [53], NCBO BioPortal [54], and GitHub [47]. In addition, we also developed a dedicated project website [46]. Note that the most up-to-date ontology file is always accessible at: http://purl.obolibrary.org/obo/ncro.owl.

The initial focus of the NCRO project is on small regulatory ncRNAs; on the next stage of development we will move to other ncRNA terms and associated relations, using the high-level placeholders that are already defined in the NCRO ontology.

## Endnotes

^1^ For instance, off-target effects are represented as the realization of dispositions. We will focus on processes related to natural biology, or on processes intended to modulate natural biology like in therapeutic or experimental use of ncRNAs.

^2^http://purl.obolibrary.org/obo/NCRO_0000229.

^3^http://purl.obolibrary.org/obo/IDOMAL_0000267.
